# Geographical inequalities in dementia diagnosis and care: A systematic review

**DOI:** 10.1016/j.inpsyc.2025.100051

**Published:** 2025-06

**Authors:** Clarissa Giebel, Megan Rose Readman, Abigail Godfrey, Annabel Gray, Joan Carton, Megan Polden

**Affiliations:** a Department of Primary Care and Mental Health, University of Liverpool, UK; b NIHR Applied Research Collaboration North West Coast, Liverpool, UK; c Department of Psychology, Lancaster University, Lancaster, UK; d Department of Health Research, Lancaster, UK

**Keywords:** Dementia, Geographical inequalities, Rural, Service access, Diagnosis

## Abstract

**Background:**

People with dementia can be disadvantaged in accessing health and social care services for diagnosis and care depending on where they live (including rural vs suburban vs. urban; postcode; country). Without an existing comprehensive synthesis of the evidence to date, the aim of this systematic review was to explore the evidence on geographical inequalities in accessing services for dementia diagnosis and care.

**Methods:**

Five databases were searched in June 2024, including studies conducted in any country, published from 2010 onwards, and in English or German. Titles and abstracts, and then full texts, were screened by at least two reviewers each. Any discrepancies were resolved in discussion with a third reviewer. Data were extracted by two researchers and synthesised narratively.

**Results:**

From 1321 studies screened and 49 full texts read, 32 studies were included in the final review. Most studies were conducted in the US, followed by the UK. Geographical inequalities in dementia are most often evidenced in relation to availability and suitability of services in different regions within a country, or a lack thereof. People with dementia residing in rural areas often experience challenges in receiving a timely diagnosis and accessing health and social care. No research has addressed geographical inequalities in accessing residential care. Innovative models on improving efficiency and quantity of diagnosis rates in rural Canada and Australia emerged.

**Conclusions:**

Health and social care services in rural areas need to be increased and made more suitable to the needs of people with dementia. More research needs to explore inequalities experienced by people with rarer forms of dementia. National strategies to overhaul the health and social care system need to focus on the rurality issue and recommend strategies to improve service access.

## Introduction

With over 55 million people living with dementia worldwide (WHO, 2022), the condition has become a major global public health concern. In light of a lack of a cure, getting the correct diagnosis and support timely remains vital to support people with dementia and their families as best as possible. However, there are many barriers to accessing a correct and timely diagnosis and adequate post-diagnostic care [22], [40], [47].

Geographic inequalities are of particular importance to people living with dementia and their carers when trying to access health and social care services. This can involve both receiving a diagnosis in the first place and being able to access suitable and timely care and support, including General Practitioners (GP), memory clinics and post-diagnostic support groups, day care, home care, and care homes. Unfortunately, many people with dementia and carers appear to experience varied difficulties based on location, which may include rural or urban settings, but also individual streets and postcodes within a city [20], [26], [31], [32]. Furthermore, geographical inequalities can be more global. It is known that lower- and middle-income countries (LMIC) such as Colombia, Brazil, and Sri Lanka tend to have reduced health care availability in general [10], [38], which is further pronounced for dementia [37]. Considering that the majority of people living with dementia live in LMICs, the notion of global geographical inequalities is equally relevant to be explored in order to highlight the areas that clinical practice and structural placement of services might be able to address to develop more equitable access to health and social care.

To date, it appears that only one systematic review has focused on addressing one element of geographical inequalities in dementia. Reviewing 38 studies, Arsenault-Lapierre et al. [3] reported rural and urban differences in the quality of dementia care by setting, reporting increased hospitalisations and mortality, reduced GP visits, and less usage of home care services in people with dementia residing in rural areas compared to urban areas. Studies were restricted to English and French and included until mid 2021. Moreover, a growing number of systematic reviews have specifically focused on rural dementia diagnosis and care over the past decade and more [41], [28], [36]. Whilst rurality is one factor of geographical aspects of living, other factors that are important to consider are regionality, postcode, as well as cross-country comparisons, which the stated reviews to date have not focused on.

The aim of this systematic review was to review and synthesise the evidence on geographic inequalities in dementia diagnosis and care both within and across countries, including the availability and accessibility of services. Findings from this review can help clinicians to identify where services for dementia, including diagnostic services such as memory clinics, might not reach the population sufficiently and how this could be addressed.

## Methods

### Search strategy

Five databases (Pubmed, PsycINFO, CINAHL, Scopus, Web of Science) were searched in June 2024 for empirical research studies published from 2010 onwards. Only empirical research studies collecting primary data, published since 2010 in English or German, focusing on any forms of geographical inequalities in dementia diagnosis or care, conducted in any country, were included. We included evidence from 2010 – 2024 given changes in national dementia strategies and potential changes to health care infrastructures, to ensure more recent evidence is included. Literature reviews, commentaries, theses, or poster abstracts were excluded, and studies focusing not on geographical inequalities in dementia diagnosis or care. One researcher (MR) searched the five databases and downloaded the search results.

### Screening

Each title and abstract of retrieved records were assessed by two research team members against inclusion criteria and excluded articles that did not meet inclusion criteria in Stage 1. The task was shared among five researchers, ensuring that each title was assessed by at least two reviewers (CG, MP, MR, AG, AG). The selected records were read in full text in Stage 2 independently by two researchers (AG, AG), and articles that meet the inclusion criteria were included in the review. Any discrepancies at stage 1 or 2 were resolved in discussion with the wider research team.

Papers were excluded based on the following criteria: No peer-reviewed primary data (i.e. letter to the editor, commentary, editorial, thesis, systematic review, scoping review, meta-analysis); Not focusing on dementia; Not focusing on geographical inequalities in dementia diagnosis and care.

### Data synthesis

Data from the included studies were extracted by two team members (AG,AG) into an excel sheet, the following data were extracted: Country; Year; Level of geographical inequality (country; rural/semi-urban/urban; city; socio-economic status; Index of Multiple Deprivation); Indicators of geographical inequalities and measurement scales.

Data were synthesised by five research team members (CG, MP, MR, AG, AG). Two team members first extracted high-level findings of each paper and with the PI clustered these into groups which had emerged from discussion with the public advisor (JC). Three researchers (CG, MR, MP) then extracted all findings for each paper, split by cluster, and narratively synthesised and compared studies with one another, grouping them within clusters if studies focused on particular outcomes, such as diagnosis, residential care, or mortality. Findings were discussed with the public advisor (JC) to discuss how these findings may be relevant for real-life living with and caring for someone with dementia.

### Public involvement

One public advisor (JC), an unpaid carer, was involved in interpreting the findings and clustering the included studies together. We’ve held two meetings with the public advisor to discuss the evidence and shape the results section, and the public advisor was reading document drafts in between. She is also contributing to a lay summary on the review for the NIHR Applied Research Collaboration North West Coast. The public advisor was reimbursed according to NIHR guidance for her contributions. .

**Fig. 1:**
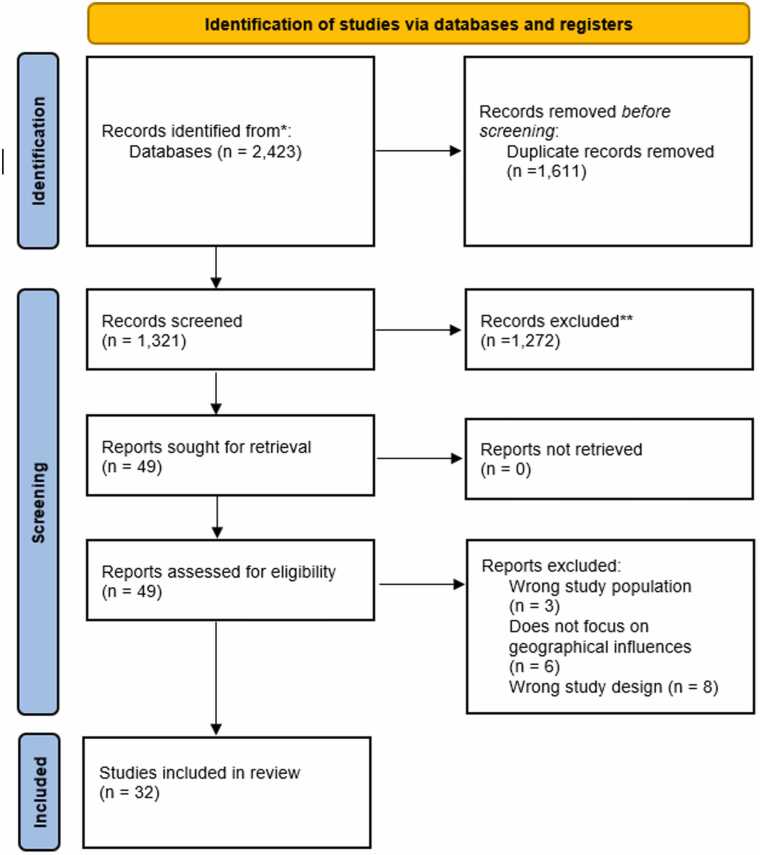
PRISMA Flowchart.

## Results

A total of 32 studies were included in the final review. Studies involved different methodologies, including analysis of routine and cohort data, surveys, semi-structured interviews, and focus groups. Studies were conducted in 11 different countries (Austria, Australia, Canada, China, Denmark, France, Italy, Ireland, Norway, UK, USA), the majority of which were conducted in the US (n = 10), followed by the UK (n = 6) and Canada (n = 6). One study was conducted across two countries – the US and Canada – without comparing inequalities across countries [45]. No study emerged that specifically compared inequalities across different countries. Table 1 provides further details about each study.

**Table 1:** Overview of included studies.

**Author (Year)**	**Country**	**Population and Sample Size**	**Methods**	**Types of geographic inequalities**	**Findings**
Abner et al.[1]	Kentucky, USA West Virginia, USA	1209,976 Medicare beneficiaries in Kentucky and West Virginia	Quantitative: Completely ecologic analysis	Rural/Urban Differences, State-Level Variations in diagnostic prevalence rates	After adjusting for various factors, ADRD prevalence in rural counties in 2013 was 11 % lower than in urban counties (95 % CI: 9 %−13 %). This lower prevalence may indicate underdiagnosis in rural areas, possibly due to the study’s ecologic design and demographic differences, such as a younger and more male population in some rural areas.
Alty et al.[2]	Tasmania, Australia	130 adults who live in Tasmania with at least 3 months of cognitive symptoms	Quantitative: Cross-sectional evaluation	Rural/Remote access to cognitive assessments and dementia diagnosis	The new single-visit cognitive clinic in Tasmania efficiently provided same-day diagnoses to 98.5 % of patients, with 36.8 % from rural/remote areas. The model reduced the wait time from referral to diagnosis by 2 months compared to national benchmarks.
Battista et al.[5]	Italy	336 Speech and language therapists	Quantitative: Survey	Barriers to Service Access	Out of 336 speech and language therapists (SLTs) surveyed, 140 had experience with primary progressive aphasia (PPA). These SLTs reported seeing an average of 3 PPA patients each over the past 24 months. SLTs with experience disclosed geographical barriers and service dysfunction as major factors limiting patient access to therapy.
Bayly et al.[6]	Canada	19 health care providers	Qualitative: Semi-structured Interviews	Inequalities in dementia care quality and educational resources in rural settings	The implementation of integrated knowledge translation (iKT) strategies with the help of a knowledge broker (KB) in two rural home care centres improved dementia care by enhancing providers' knowledge and capacity to use best practices.
Bouldin et al.[8]	USA	7436 caregivers	Quantitative: Cross-sectional observational study	Financial and health barrier between rural and urban caregivers across 10 states.	Caregiving was more common in rural areas (21.4 %, 95 % CI: 20.4 −22.5) compared to urban areas (19.0 %, 95 % CI: 18.0 −19.9; P = 0.0005). Rural caregivers faced more financial barriers (38.1 % vs. 31.0 %) but had similar health barriers compared to urban caregivers.
Brady et al.[9]	Ireland	8 general practitioners, 1 person living with dementia, 7 family members of people living with dementia	Qualitative: Semi-structured interviews	Diagnosis inequalities throughout regions	Dementia is significantly underdiagnosed in Longford-Westmeath, Ireland. Rural areas face significant gaps in dementia awareness, limited diagnostic resources, and fewer specialist services compared to urban areas.
Constantinescu et al.[13]	Alberta, Canada	16 family physicians	Qualitative: Semi-structured focus groups	Rural-Urban differences in family physicians’ challenges in providing dementia care	Rural family physicians struggle with limited knowledge about dementia symptoms, diagnostic processes, and advanced diagnostic tools. Challenges include limited access to specialist services, long wait times, and inadequate community resources for dementia care.
Cooper et al.[14]	United Kingdom	77045 patients with recorded dementia diagnosis or anti-dementia drug prescription	Quantitative: Observational cohort study	Regional variations in access to anti-dementia drugs	Northern Ireland: 81 % more likely to prescribe anti-dementia drugs than England (incidence rate ratio 1.81, 95 % CI: 1.41–2.34). Wales: 32 % less likely to prescribe compared to England (incidence rate ratio 0.68, 95 % CI: 0.55–0.82). Scotland: Prescribing rates showed a trend towards being higher than in England (incidence rate ratio 1.14, 95 % CI: 0.98–1.32).
Cotton et al.[15]	USA	10 informal caregivers residing in socially disadvantaged areas	Qualitative: Semi-structured interviews	Caregivers’ experiences with supportive services in under-resourced areas	Caregivers in socially disadvantaged areas often turn to supportive services in response to crises or unmet needs, following a sequence of seeking, initiating, and using services.
Czapka et al. (2020)	Oslo, Norway	7 migrants in Oslo engaged in transnational caring activities for their parents living with dementia.	Qualitative: Semi-structured interviews	Differences in Care Systems across caregivers’ country of residence	Migrants face significant difficulties in transnational caregiving for parents with dementia, including redefining care roles, developing new strategies, and negotiating care scope with family members in the country of origin.
Dal Bello-Haas et al.[17]	Canada	154 Rural, community-dwelling individuals with mild to moderate dementia and their caregivers.	Mixed methods: Survey and semi-structured interviews	Inequality of accessing exercise interventions for individuals living in rural areas.	50 % of surveyed patients and caregivers showed interest in telehealth-based exercise interventions. Willingness to participate in group exercise significantly predicted interest in telehealth, accounting for 24.4 % of the variance (F-statistic=16.14, p < 0.001).
Forbes et al.[18]	Canada	Nine rural dementia care networks consisting of PWD (n = 5), care partners (n = 14), and HCPs (n = 14)	Qualitative: Interviews	Inequalities in dementia care between rural and more urban areas.	Rural areas face significant barriers in dementia care due to limited community resources and integration, with caregivers relying heavily on local and personal sources for information. Persons with dementia often do not recognise their need for care information, leading to potential isolation for caregivers.
Gibson et al.[19]	Midwest USA	11 rural caregivers of persons with dementia	Qualitative: Semi-structured interviews	Access to dementia-specific services between rural and urban areas.	Rural caregivers of persons with dementia face unique challenges, including financial concerns, geographic barriers, and a lack of dementia-specific services. Despite these challenges, many caregivers found strength and support within their community, which often served as a safety net.
Haydon et al.[25]	Australia	73 people from three Aboriginal Medical Services and communities.	Qualitative: Focus groups and interviews	Providing dementia care for people living in rural and remote area	Rural caregivers of persons with dementia have different needs than urban caregivers, requiring tailored outreach and services. Effective interventions should target multiple systems to promote social change for marginalised populations.
Hicks et al.[26]	England	17 rural-dwelling older men with dementia	Qualitative: Open interview	Access to dementia care and support services	The study identified two main themes. First, rural living offered benefits like a pleasant environment and supportive networks. Second, challenges included lack of dementia awareness and physical and motivational barriers. These findings highlight how the rural environment both helps and holds back older men with dementia, leading to recommendations for promoting social inclusion.
Jørgensen et al.[29]	Denmark	Danish individuals aged 65 + from January 1, 2000, and followed those without Alzheimer’s from January 1, 2008, until diagnosis, death, emigration, or December 31, 2009.	Quantitative: Regression analysis	Distance to an Alzheimer clinic and association to Alzheimer diagnosis	Individuals diagnosed with Alzheimer’s between 2008 and 2009 had a shorter average distance to clinics (16.6 km) compared to those not diagnosed (19.1 km) (P < 0.001). Distance to clinics inversely affected diagnosis likelihood, with hazard ratios of 0.80 (95 % CI: 0.70–0.92) for 20–39 km and 0.65 (95 % CI: 0.52–0.81) for 40–59 km, indicating that greater distances were associated with fewer diagnoses.
Krutter et al.[30]	Austria	107 family caregivers	Mixed methods: Questionnaires and regression analysis	Home care nursing in a rural setting	Higher age of family caregivers and female gender of persons with dementia (PwD) are key factors influencing the use of home care nursing in rural areas.
Li et al.[31]	Illinois, USA	109 rural older adults with Alzheimer’s disease	Mixed methods: Telephone interviews and regression analysis	Unmet home care service needs	53.2 % of patients experienced unmet service needs. Unmet needs were most frequently reported for bathing (30.9 %), dressing (27.1 %), walking (26.3 %), housekeeping (32.6 %), meal preparation (22.6 %), and transportation (22.1 %). Caregivers who experienced a higher level of burden were likely to report a higher rating on unmet service needs (β = −.18, p = .06).
Loup et al.[32]	Alabama and Mississippi, USA	30 rural-dwelling dyads, individuals with dementia (n = 12) and informal caregivers (n = 18)	Qualitative: Semi-structured interviews	Resource needs	Dyad #2: difficulties driving due to troubles getting PWD in the car and needing to travel to a distant physical therapist two times each week. Dyad #5: need to increase PWD’s mobility beyond his house to get him to care appointments. Dyad #4: takes a day to complete grocery shopping due to driving distance and physical demands.
McPherson [33]	Rural Washington, USA	17- informal caregivers (n = 12), community members (n = 5)	Quantitative: Survey	Patient-caregiver dyad support groups	47 % responded neutral that there were support services for people with early stage AD. 47 % agreed that there were support services for informal caregivers of people with early stage AD. 4 participants stated that they do not know of any early stage AD support services. 70.6 % were neutral on the location of services. Information about services was the most recognised barrier to accessing support (29.4 %). Responded neutral that location of services, transportation to services were barriers to accessing support services.
Morgan et al.[34]	Saskatchewan, Canada	11 directors of care and 4 family caregivers	Mixed methods: Focus groups and survey	Barriers to use of formal services	Stigma of dementia due to it being associated with mental illness and the generation that it is hitting. Lack of privacy and anonymity due to living in a rural area where one’s activities were likely to be observed and discussed. Lack of information about the availability of services. Access to experts in assessment and management was limited in rural areas. Distance to services was a barrier. Limits on the amount and types of service that could be obtained in a given time period were also issues. The cost of home services was a deterrent.
Ouvrard 2020 [35]	France	3431participants aged 65 years or over	Quantitative: Epidemiological cohort study.	Impact of geographical deprivation on risk of dementia	There was no association between geographical deprivation and risk of dementia, except for the participants in the Q3 (second most geographically deprived) compared to those in the Q1 (least geographically deprived) (HR=1.35; 95 % CI: 1.06, 1.72).
Teel [42]	A mid-western state, USA	19 primary care providers	Qualitative: Semi-structured interviews	Diagnosis and treatment	Several PCPs pointed out difficulties in trying to access or coordinate consultation, either due to infrequent visits by consultant to the PCP’s rural area, the extreme distances patients had to travel to the specialist’s office, or the delays in getting appointments. Difficulties in communicating with the family and limited community resources.
Thorpe et al.[43]	USA	1186 community-dwelling older male veterans with dementia and their female informal caregivers.	Quantitative: Observational cross-sectional	Preventable hospitalisations among community-dwelling veterans with dementia	Compared to those in large metropolitan counties, the odds of having an ACSH (ambulatory care sensitive hospitalisations) were 1.99 times greater for care recipients living in noncore counties (12.8 % vs 22.6 %; OR=1.99, p < .01) Odds of ACSH did not differ significantly between large metropolitan counties, small metros, and micropolitan counties. The relationship between county rurality and non-ACSH was not statistically significant.
Vipperman et al.[45]	Appalachia	124 family caregivers of people living with dementia.	Qualitative: Telephone interviews	Barriers to service use among dementia family caregivers	Caregivers’ capability of seeking services had a marginal effect on support service use (IRR=0.71, p = 0.07). Caregivers who experienced financial barriers (OR=0.001, p < .01) and those who were reluctant to use services (OR=0.02, p < .01) had significantly lower odds of using personal care services.
Watson et al.[47]	England	142,302 people living with dementia.	Quantitative: Observational study	Inequalities in primary and secondary healthcare	People living with dementia from rural areas have greater use of healthcare services more closely associated with negative health outcomes, including less frequent GP contact and medications, and greater use of secondary healthcare.
Watson et al.[48]	England	142,302 people living with dementia	Quantitative: Observational longitudinal study	Social and spatial inequalities	People with rural GP practices had significantly fewer GP observations than urban (IRR: 0.909; 0.900 −0.919). In later-onset dementia, A&E attendances were more likely among people living with dementia with rural GPs (OR: 1.204; 1.156 −1.253), but emergency hospital admission spells were less likely (OR: 0.820; 0.787 −0.855). Compared to the Northeast GP region, people living with dementia registered with GPs in other regions had significantly fewer GP contacts but more non-dementia medications.
Watson et al.[46]	England	142,340 people living with dementia with at least 2 years of post-diagnosis follow-up data	Quantitative: Observational longitudinal study	Mortality risk	Compared to the Southeast Coast region, mortality risk was greater in the South Central (HR: 1.23; 1.17 −1.29), Southwest (HR: 1.17; 1.11 −1.23), Northeast (HR: 1.10; 1.03 −1.16) GP regions.
Wu et al.[49]	Great Britain	1547 people with dementia and 1283 carers	Quantitative: Observational cross-sectional	Living well with dementia	Mean scores for living well were similar across urban and rural areas. There was no substantial difference in living well indicators across urban and rural areas.
Yin et al.[50]	China	161 counties and districts	Quantitative: Observational longitudinal study	Dementia mortality	Mortality rates were significantly higher in the east (rate ratio 2.28) compared with the north. Dementia mortality decreased by 15 % in urban areas but increased by 24 % in rural areas.
Zakarias et al.[51]	Denmark	5516 patients who had been diagnosed in 2015	Quantitative: Observational cross-sectional	Diagnostic quality	The age and sex-standardised national prevalence rate of registered dementia diagnoses was 3.0 %, ranging from 2.5 % to 3.6 % in the five regions. The proportion of patients who were registered with a specific dementia diagnosis at their first diagnosis varied from 45.3 % to 75.5 % in the regions. The proportion of patients diagnosed at a dementia specialist department ranged from 60.9 % to 90.5 % across the five regions.

### Direct comparisons of geographical inequalities in diagnosis and care

Eleven studies directly compared geographical inequalities in diagnosis and care across different geographical regions, nine using quantitative data, and two using qualitative methodologies. These included a focus on diagnosis rates and subtype diagnoses, anti-dementia drug prescription, health care usage, and mortality risks.

There is limited evidence to date on geographical inequalities in dementia diagnosis. Evidence by Abner et al. [1] is the only to showcase different overall diagnosis rates by region, with 11 % lower diagnosis rates in rural compared to urban counties (in the US). This is complemented by findings from Denmark, showing that the proportion of people receiving a specific subtype diagnosis varied between 45.3 to 75.5 % across regions, with the capital region of Copenhagen having the lowest subtype diagnosis proportion and the southern region of Denmark the highest [51].

Four studies directly compared geographical inequalities in relation to health care utilisation. Focusing on anti-dementia drug prescription rates, Cooper et al. [14] evidenced lower anti-dementia drug prescription rates in more deprived patients with dementia in England compared to Scotland, Wales, and Northern Ireland, based on the Index of Multiple Deprivation (IMD) determining neighbourhood, not individual, deprivation. In the latter three countries, prescribing rates were not associated with socio-economic status and deprivation quintile. Overall, regardless of socio-economic status, the authors noted significantly higher prescribing rates in Northern Ireland compared to England, and Wales. In contrast, Watson et al. [47] noted the reverse, whereby people from more disadvantaged backgrounds had higher rates of anti-dementia drug prescriptions. Furthermore, people with dementia residing in rural areas had reduced levels of GP visits compared to those residing in more urban areas in England [47], whilst rurality was linked to higher rates of A&E attendance in people with late-onset dementia. Similarly, utilising 5-year health care trajectory data from England, Watson et al. [48] reported how people with dementia from less deprived backgrounds (as measured via the IMD) experienced more later increases in health care use and more stable GP contact, compared to people with dementia from more deprived backgrounds. The only evidence from the US directly comparing health care access in different regions evidenced greater likelihood of ambulatory care sensitive hospitalisations in veterans with dementia living in rural counties as opposed to metropolitan areas [43], thereby supporting evidence from England by Watson et al. [47] regarding hospitalisation rates by geographical living location. It was not feasible to discern whether rates varied due to availability or access barriers.

Geographic variations in mortality risk in dementia were evidenced in two studies. Using electronic health records, Watson et al. [46] reported higher levels of mortality risk in people with dementia living in more deprived areas (as measured via the IMD), and living in the North East, South Central, and South West of England. No other study has compared geographical inequalities in mortality risk in England, and Yin et al. [50] provides the only other evidence in the field but focusing on China. Specifically, using data from the China Mortality Surveillance System, the authors reported increased levels of mortality of people with dementia in rural areas (by 24 %) with reduced mortality in urban areas. Moreover, mortality was significantly higher in the East of the country compared to the North. The authors propose that these variations may in part be due to service availability. Specifically, suggesting that the declining mortality trend in urban areas may reflect better health infrastructure and institutional care as well as palliative and hospice care services compared to rural areas. Given the lack of evidence across and within countries, no comparisons can be drawn. However, findings indicate that geographical variations exist and require further research.

Besides service access, only one study directly compared the impact of socio-economic deprivation and geographic living location on well-being in dementia. Using cohort data, Wu et al. [49] showed that people with dementia from more disadvantaged backgrounds (as measured by the IMD and thus postcode) who are living with a carer in Great Britain had poorer quality of life, life satisfaction and wellbeing compared to people with dementia from more advantaged backgrounds.

No study qualitatively compared experiences between rural and urban settings.

### Health care services – availability, access, and use

#### Diagnosis

Obtaining an accurate and timely diagnosis is crucial in order to receive the right care as soon as possible (which may be pharmacological, non-pharmacological, or a mix of both), and to plan ahead for specific symptom deterioration, yet geographic location often plays a crucial role in obtaining a diagnosis due to diagnostic service availability. Abner et al. [1] found that diagnostic prevalence was 11 % lower in rural areas of the U.S. compared to urban areas, likely due to reduced access to diagnostic services and care in these communities. Brady et al. [9] highlighted that the impact of service availability, due to geographical location, on obtaining a diagnosis is not unique to the U.S. Specifically, GPs in Ireland raised concerns about access to specialised dementia services, particularly during the diagnosis and assessment stages, which contributes to delays in receiving timely diagnoses. Similarly, primary care physicians, in mid-western America, highlighted difficulties in accessing and coordinating diagnostic consultations, either due to infrequent visits by consultants to rural areas, or the extreme distances people with dementia had to travel to the specialist’s office [42]. Echoing the opinions of primary care professionals, Jørgensen et al. [29] observed a link between geographical distance to Alzheimer’s clinics in Denmark and the likelihood of receiving an Alzheimer’s diagnosis. Increased distance to healthcare providers was linked to reduced rates of Alzheimer’s diagnoses. Geographical variations also occurred in dementia subtype diagnosis. Indeed, Zakarias et al. [51] observed that in Denmark the proportion of people receiving a specific dementia subtype diagnosis varied between 45.3 to 75.5 % across regions with the capital region of Denmark having the lowest subtype diagnosis proportion and the southern region of Denmark having the highest subtype diagnosis proportion.

Consistent with the above evidence, Alty et al. [2] acknowledged that unequal access to cognitive assessments is a significant barrier to timely diagnosis in rural or remote areas, whether related to availability or access issues. They proposed a solution involving ‘one-stop’ cognitive clinic models, examining this approach in a remote area of Australia. In their study, 98.5 % of 130 adults received a same-day diagnosis, indicating that ‘one-stop’ models may effectively reduce health inequalities in rural areas by facilitating timely diagnosis.

#### Continuous health care

Access to continued engagement with health care appears to be influenced by geographical factors. For example, Watson et al. [47] found that people with young-onset dementia with rural GP practices had fewer GP observations then people with dementia in urban areas. Moreover, in later-onset dementia, people with rural GPs had higher A&E attendances but interestingly had lower emergency hospital admissions. This finding may in part be explained by Thorpe et al. [43] who discusses that people with dementia in rural areas face additional challenges in receiving timely and effective ambulatory care compared to those in urban areas. Moreover, Loup et al. [32] identified substantial commutes to health care appointments as a barrier to health care engagement in people with dementia in rural localities, suggesting availability barriers, whilst evidence often does not allow to identify whether using services is subject to a lack of availability or other factors influencing access.

Geographical variability in access to continued health services is not constrained to GP engagement. By interviewing health and social care professionals, Forbes et al. [18] found that people with dementia in rural communities of Ontario, Canada, may receive less routine follow-up at home due to longer travel times between clients. Moreover, Constantinescu et al. [13] showed that people with dementia in rural Canada have limited access to specialist geriatric care, not only due to lack of service provision but also due to lack of rural family physician knowledge. This was echoed by Cotton et al. [15], who observed that limited access to support in Midwestern US may be due to increased difficulty in finding an appropriate service/medical professional in rural areas compared to urban areas.

### Social care services – availability, access, and use

Ten studies in this review have examined geographical inequalities in social care settings, including home care and community-based support services. Many of these studies highlighted that remote areas and regions with higher levels of deprivation face significant challenges, negatively affecting the availability, accessibility, and overall effectiveness of social care services. No study focused on geographical inequalities in accessing residential care.

#### Home care settings

Four studies explored geographical inequalities in home care services ([30], [31], [6]; Rabarison et al., 2018). Krutter et al. [30] found that in rural Austria, geographical location was not a significant barrier to the availability and use of home care services. This suggests that home care services were not limited in rural areas in Australia, although this finding is based on one study. Instead, factors including carer age, gender, employment status, and education were identified as enablers of home care utilisation. In contrast, Li et al. [31] examined unmet home care needs for people with Alzheimer’s in rural areas by collecting perspectives from 109 unpaid carers. Their findings suggest that individuals who accessed more formal services had fewer unmet needs overall, highlighting the importance of expanding home care services in rural areas where formal care options are limited.

Consistent with Krutter et al. [30] and Li et al. [31], Rabarison et al. (2018) explored geographical inequalities in home care settings and investigated the economic value of informal caregiving for people with dementia across 38 US states. Their findings showed that informal caregiving was more prevalent in rural areas than in urban ones. Consistent with Li et al. [31], this may point to a greater dependence on unpaid carers and home care services in rural regions due to limited access to formal care services. However, no significant differences were found in terms of health-related barriers, as carers in both rural and urban settings faced similar challenges.

Whilst improving knowledge in home care staff may not necessarily overcome rural inequalities in access, Bayly et al. [6] reported that embedding integrated knowledge translation in two rural home care centres in Canada improved care providers’ knowledge of dementia. This can lead to improved care delivery in rural regions, and is the only solution evidenced in the included literature on improving care in areas where services may be sparse and knowledge may be limited.

#### Community-based support services

Seven studies have highlighted geographical inequalities in the provision of social care services. McPherson [33] conducted a needs assessment survey in rural U.S. towns, revealing that many participants lacked information about early-stage support groups and faced barriers to accessing them. Although these support groups could have addressed their unmet needs, the main obstacle seemed to be the lack of information provided to people with dementia about the availability of local support services and how to access them, rather than the lack of services themselves.

Some benefits were noted in Hicks et al. [26] study regarding dementia support for men in rural areas. The study found that rural communities offered various structured activities, and informal rural networks helped participants stay engaged and connected. However, challenges related to rural living were also discussed, such as difficulties navigating the rural landscape due to reduced mobility, leading to social isolation. Additionally, limited transportation options increased reliance on carers to help people attend and access dementia support services. Gibson et al. [19] highlighted additional benefits of rural life, including strong community ties, whilst also identifying substantial geographical inequalities. Rural carers faced unique challenges, such as financial concerns, geographic barriers, and a lack of dementia-specific services. When considering access to specialist services for people living rarer dementia subtypes, Battista et al. [5] observed that in Italy people with Primary progressive aphasia (PPA) may not have access to specialist speech and language services due to their geographical distance from service providers. This was the only study though that explored the impact of geographical inequalities on people with rarer dementia subtypes.

Consistent with findings from McPherson [33], Hicks et al. [26], Battista et al. [5], and Gibson et al. [19], Haydon et al. [25] highlighted the need for tailored, widely available and accessible social support in rural areas. Haydon et al. [25] found that disparities existed between biomedical and culturally appropriate models of dementia care, disproportionately affecting people in rural and remote areas. This aligns with Cotton et al. [15], who identified a demand for more specialised dementia services in underserved areas, both for people living with dementia and their caregivers. Their findings suggested that as dementia progresses, individuals’ needs evolve, requiring a range of specialised services that are often less available in underserved communities, thereby widening inequalities. These inequalities were found to extend to digital health services and telehealth with Dal Bello-Haas et al. [17] finding barriers to delivering telehealth in rural regions. However, despite these barriers, there was high acceptability for telehealth interventions suggesting their potential to improve service accessibility, particularly for people in remote locations.

#### Financial background

Eleven studies reported on the financial impacts of geographical inequalities for people with dementia. Whilst financial barriers to accessing care are not unique to people with dementia and unpaid carers living in rural settings, financial barriers can be pronounced further in those living in remote settings. Bouldin et al. [8] reported that rural carers across 10 US states experienced difficulties in being able to afford medical care for people with dementia significantly more frequently than urban carers. This was based on either an annual household income of less than $25,000 or having been unable to visit a doctor when needed. Cotton et al. [15] furthermore reported how limited household income and personal finances in unpaid carers from socio-economically disadvantaged backgrounds in the US could affect service selection, leading to selection of less suitable, geographically closer services. Similar findings have emerged from Austria. Using regression modelling, Krutter et al. [30] reported that higher household income was linked to increased levels of home care service usage.

This was corroborated and further expanded on by Czapka and Sagbakken [16] who interviewed trans-national carers of people with dementia. Particularly Polish carers were often struggling financially to cover the costs of caring, in a society where caring tends to be provided by women in the family. Where people with dementia and their families/carers lived in different countries, this could place additional burden and difficulties on the caring relationship, with a need for being able to access and afford external care services. High service costs in general were noted in other countries also, such as the UK, US and Canada in rural Appalachia [19], [45], leading many people to not access care services. Whilst not identifying how rurality and geographical location affected this, Battista et al.’s (2023) survey showed that high service costs impeded some people with primary progressive aphasia from accessing speech and language therapy for their dementia symptom.

Only one included study focused on the link between personal or community socio-economic background and risk of dementia (Ouvrard 2020 [35]). The authors showed that higher levels of personal socio-economic deprivation were linked to significantly higher dementia risk. However, community index levels of deprivation were not found to be associated with dementia risk.

From a service access provision point of view, only two studies explored the link between area-level socio-economic deprivation and anti-dementia medication prescription and wider healthcare use and access. Utilising over 77,000 patient records, people with dementia from the least deprived backgrounds as measured by the IMD were 25 % more likely to receive anti-dementia medication than those from the most deprived backgrounds in the UK [14]. Furthermore, prescribing rates were significantly lower in people from lower socio-economic backgrounds in England compared to those living in Wales, Scotland, and Northern Ireland. This trend in experiences for people with dementia from more deprived backgrounds was also evidenced when examining health care records on GP visits, accident and emergency attendance, and hospital admissions. Contrary to Cooper et al. [14], using Clinical Practice Research Datalink (CPRD) data from GP settings, Watson et al. [47] evidenced higher likelihood of dementia medication prescription in people with late-onset dementia (aged 65 +) from the most deprived backgrounds, compared to those from the least deprived backgrounds. Both studies used CPRD data, with Watson et al. [47] including data up to 2020 and thus more recent data than Cooper et al. [14]. In addition, Watson et al. [47] coded prescription rates into dementia and non-dementia, whereas Cooper et al. [14] coded drug-initiation rate for donepezil, rivastigmine, galantamine, and memantine. This may explain the difference in the link between socio-economic background and anti-dementia medication prescription.

### Quality ratings

Quality ratings of included studies are detailed in Table 2, Table 3. All studies were rated between 70 % and 95 % based on either the CASP tool for qualitative studies or the AXIS tool for cross-sectional studies. Thus, no study appeared to have poor methodological reporting.

**Table 2:** Quality assessment of qualitative studies based on CASP.

	**Q1**	**Q2**	**Q3**	**Q4**	**Q5**	**Q6**	**Q7**	**Q8**	**Q9**	**Q10**	**Score**
Bayly et al.	YES	YES	YES	YES	YES	NO	NO	YES	YES	YES	70 %
Brady et al.	YES	YES	YES	YES	YES	YES	YES	YES	YES	YES	90 %
Constantinescu et al.	YES	YES	YES	YES	YES	NO	YES	YES	YES	YES	80 %
Cotton et al.	YES	YES	YES	YES	YES	NO	NO	YES	YES	YES	70 %
Czapka et al.	YES	YES	YES	YES	YES	NO	YES	YES	YES	YES	80 %
Forbes et al.	YES	YES	YES	YES	YES	NO	NO	YES	YES	YES	70 %
Gibson et al.	YES	YES	YES	YES	YES	YES	NO	YES	YES	YES	80 %
Haydon et al.	YES	YES	YES	YES	YES	YES	YES	YES	YES	YES	90 %
Hicks et al.	NO	YES	YES	YES	YES	YES	YES	YES	YES	YES	80 %
Li et al.	YES	YES	YES	YES	YES	NO	NO	YES	YES	YES	70 %
Loup et al.	YES	YES	YES	YES	YES	YES	NO	YES	YES	YES	80 %
Morgan et al.	YES	YES	YES	YES	YES	YES	YES	YES	YES	YES	90 %
Teel et al.	YES	YES	YES	YES	YES	YES	NO	YES	YES	YES	80 %

**Note.** Q1. Was there a clear statement of the aims of the research? Q2. Is a qualitative methodology appropriate? Q3. Was the research design appropriate to address the aims of the research? Q4. Was the recruitment strategy appropriate to the aims of the research? Q5. Was the data collected in a way that addressed the research issue? Q6. Has the relationship between researcher and participants been adequately considered? Q7. Have ethical issues been taken into consideration? Q8. Was the data analysis sufficiently rigorous? Q9. Is there a clear statement of findings? Q10. How valuable is the research? “Yes” coded as 1; “no” and “can't tell” coded as 0.

**Table 3:** Quality assessment of quantitative studies based on AXIS.

	**Q1**	**Q2**	**Q3**	**Q4**	**Q5**	**Q6**	**Q7**	**Q8**	**Q9**	**Q10**	**Q11**	**Q12**	**Q13**	**Q14**	**Q15**	**Q16**	**Q17**	**Q18**	**Q19**	**Q20**	**Score**
Abner et al.	YES	YES	YES	YES	YES	YES	CAN'T TELL	YES	YES	YES	YES	YES	CAN'T TELL	YES	YES	YES	YES	YES	NO	YES	90 %
Alty et al.	YES	YES	YES	YES	YES	YES	NO	YES	YES	YES	NO	YES	NO	YES	YES	YES	YES	YES	NO	CAN'T TELL	80 %
Bouldin et al.	YES	YES	YES	YES	YES	YES	NO	YES	YES	YES	YES	YES	NO	YES	YES	YES	YES	YES	NO	CAN'T TELL	85 %
Cooper et al.	YES	YES	YES	YES	YES	YES	NO	YES	YES	YES	YES	YES	NO	YES	YES	YES	YES	YES	NO	YES	90 %
Ellajosyula et al.	YES	YES	YES	YES	YES	YES	YES	YES	YES	YES	YES	YES	NO	YES	YES	YES	YES	YES	NO	YES	95 %
Jørgensen et al.	YES	YES	YES	YES	YES	YES	NO	YES	YES	YES	YES	YES	NO	YES	YES	YES	YES	YES	NO	NO	85 %
Krutter et al.	YES	YES	YES	YES	YES	YES	NO	YES	YES	YES	YES	YES	NO	YES	YES	YES	YES	YES	NO	YES	90 %
McPherson [33]	YES	YES	NO	YES	NO	YES	YES	YES	NO	NO	YES	YES	YES	NO	CAN'T TELL	YES	YES	YES	CAN'T TELL	YES	70 %
Ouvrard et al.[35]	YES	YES	YES	YES	YES	YES	NO	YES	YES	YES	YES	YES	NO	YES	YES	YES	YES	YES	YES	YES	95 %
Thorpe et al.	YES	YES	YES	YES	YES	YES	NO	YES	YES	YES	YES	YES	NO	YES	YES	YES	YES	YES	CAN'T TELL	CAN'T TELL	85 %
Vipperman et al. [49]	YES	YES	YES	YES	YES	YES	NO	YES	YES	YES	YES	YES	NO	YES	YES	YES	YES	YES	NO	YES	90 %
Watson et al.[47]	YES	YES	YES	YES	YES	YES	NO	YES	YES	NO	YES	YES	NO	YES	CAN'T TELL	YES	YES	YES	NO	YES	80 %
Watson et al.[48]	YES	YES	YES	YES	YES	YES	NO	YES	YES	YES	YES	YES	NO	YES	YES	YES	YES	YES	NO	YES	90 %
Watson et al.[46]	YES	YES	YES	YES	YES	YES	YES	YES	YES	YES	YES	YES	NO	YES	YES	YES	YES	YES	NO	YES	95 %
Wu et al.	YES	YES	YES	YES	YES	YES	CAN'T TELL	YES	YES	YES	YES	YES	NO	YES	YES	YES	YES	YES	NO	YES	90 %
Yin et al.	YES	YES	YES	YES	YES	YES	CAN'T TELL	YES	YES	YES	YES	YES	NO	YES	YES	YES	YES	YES	NO	NO	85 %
Zakarias et al.	YES	YES	YES	YES	YES	YES	YES	YES	YES	YES	YES	YES	NO	YES	YES	YES	YES	YES	NO	YES	95 %

**Note.** Q1. Were the aims/objectives of the study clear? Q2. Was the study design appropriate for the stated aim(s)? Q3. Was the sample size justified? Q4. Was the target/reference population clearly defined? (Is it clear who the research was about?) Q5. Was the sample frame taken from an appropriate population base so that it closely represented the target/reference population under investigation? Q6. Was the selection process likely to select subjects/participants that were representative of the target/reference population under investigation? Q7. Were measures undertaken to address and categorise non-responders? Q8. Were the risk factors and outcome variables measured appropriate to the aims of the study? Q9. Were the risk factor and outcome variables measured correctly using instruments/measurements that had been trialled, piloted or published previously? Q10. Is it clear what was used to determine statistical significance and/or precision estimates? (e.g. p-values, confidence intervals) Q11. Were the methods (including statistical methods) sufficiently described to enable them to be repeated? Q12. Were the basic data adequately described? Q13. Does the response rate raise concerns about non-response bias? Q14. If appropriate, was information about non-responders described? Q15. Were the results internally consistent? Q16. Were the results presented for all the analyses described in the methods? Q17. Were the authors’ discussion and conclusions justified by the results? Q18. Were the limitations of the study discussed? Q19. Were there any funding sources or conflicts of interest that may affect the authors' interpretation of the results? Q20. Was ethical approval or consent of participation attained? Yes” coded as 1; “no” and “can't tell” coded as 0.

## Discussion

This is the first systematic review to synthesise the evidence on geographical inequalities beyond rurality and urban-space living in dementia focusing on not only quality of dementia care, but also on diagnosis, access of health and social care services, and mortality in dementia. Advancing Arsenault-Lapierre et al. [3] review, this review highlights that the primary impact of geographical inequalities in dementia is experienced on the availability and suitability of available services, with accessing the right dementia care further being impeded by a lack of financial means and transport availability.

Evidence from 11 countries has highlighted difficulties in accessing the right health (GP, hospital) and social care (home care, community-based services) services in dementia. Overall, living in rural and remote areas was found to hinder accessing care and diagnosis due to the lack of available, or good quality and suitable, services (i.e. Boudard et al., 2018; [16]) and distance to and time required to access the services. For example, specialist diagnostic [1], [2], [9] and palliative and hospice care services [50] services appear to be specifically lacking in rural compared to urban communities. Tackling this issue will require a greater focus on establishing a digital infrastructure to access services remotely in addition to increasing in-person service provision and thus reducing required transport and time. As research conducted particularly during and since the COVID-19 pandemic has highlighted, only accessing remote (digital/ telephone) service does not provide sufficient care and support and is not feasible for certain types of care requirements in older adults and people living with dementia [12], [44]. To facilitate improved service provision, national dementia strategies need to be in place to provide governments and service providers with the necessary priority to deliver more accessible care regardless of living location. In a recent review of 15 national dementia strategies, having only included those published in English or French, Godard-Sebillotte et al. [24] reported that only six of 15 strategies had mentioned rurality as a barrier to accessing care (Australia, Canada, Denmark, Germany, Greece, USA), whilst only two strategies (Australia and the USA) provided objectives to address this issue.

On a service-provider level, two innovative solutions have emerged and were identified in this review that helped address rural inequalities and increase diagnosis rates and knowledge sharing. Alty et al. [2] and Bayly et al. [6] evidenced improved and more efficient diagnosis rates in rural areas in Tasmania, Australia and positive impacts of knowledge transfer in two home care settings in Canada on the home care workforce and people with dementia and carers accessing the service, respectively. These service models provide positive potentials for improving diagnosis rates and reducing need for travel, by having a single, time-efficient, setting that provides a diagnosis within one day, as opposed to the need to travel to repeated assessments. Moreover, improving the knowledge base in rural care providers is critical to improve quality of care, especially for people with rarer subtypes of dementia who can struggle getting the support they need in low-populated areas, where there are even fewer cases of the same subtype diagnosis.

Increasing knowledge is equally relevant for people with dementia and unpaid carers in order to improve access to relevant services in more rural regions. However, lack of knowledge about existing services and steps to take after the diagnosis is not restricted to people residing in more rural regions [11]. Many people with dementia and their carers complain about the lack of clear pathways in dementia and who to contact, which services are available, and what financial support may be available to access relevant services [39]. Thus, another solution to increasing service access, and thereby addressing inequalities in access, would be general awareness raising of dementia. This can take place from the point of diagnosis at the memory clinic, or via more public health measures and wider initiatives to increase knowledge. One such method could be via social game play, such as via the Dementia Inequalities Game, which has proven to significantly improve knowledge about dementia and inequalities in the general population and in University students [21], [23].

Linked to inequalities in accessing suitable services, only one study focused on geographical inequalities on subtype-related care [5] and one study focused on subtype diagnosis rates by region [51]. Whilst rates of subtype diagnoses were lowest in the Danish capital Copenhagen [51], people with a diagnosis of PPA in Italy were found to experience difficulties in accessing suitable support for their symptoms and needs, requiring further travel distances to access the right care [5]. Considering the higher frequency of other subtypes such as Lewy Body or front-temporal dementia [27], it is surprising to witness only one included study reporting the geographical inequality impacts on people with or carers for someone with a rarer subtype of dementia, which focused on PPA. Alzheimer’s disease dementia is the most common subtype, with different subtypes experiencing different needs and requirements for care. Future research ought to explore the service availability and suitability for people with rarer dementia subtypes across different countries.

Geographic inequalities were also linked to financial barriers in 11 studies. Several studies for example utilised the IMD as a neighbourhood measure of socio-economic deprivation, mostly reporting those residing in poorer areas to face greater barriers to accessing care (and risk of developing dementia). The only exception was research into anti-dementia medication by neighbourhood deprivation, with opposing findings [14], [47] from the UK likely the result of different data capture points and how prescription rates were measured. Besides IMD, financial barriers in more rural areas can present themselves by having to pay for travel costs to reach available or suitable services, with limitations in public transport (e.g. [8], [30]). With limited service availability, rurality can also be linked to having to pay for more suitable services instead of using those closer by (e.g. [15]). It is to be noted that no single study has emerged from LMICs, where accessing any form of medical care is often impeded by a lack of funds, and general lack of sufficient and adequate freely available healthcare infrastructure [4], [7]. Thus, evidence to date strongly highlights how people with dementia and their carers in high-income countries face substantial financial barriers linked to their living location, and future research needs to explore this issue in detail in LMICs to provide a more holistic overview of this issue. This should also be done by comparing experiences in different countries within a study, which to date does not appear to have been evidenced based on the studies included. However, this may have been because multi-country studies perhaps fail to use ‘geographical inequalities’ in their key search terms.

## Limitations

Although this review benefitted from double screening of all titles/abstracts and full texts and included evidence from five evidence bases, the studies included were only published in English and German. This may have left out some evidence from LMICs for example such as Vietnam or Colombia that was published in other languages.

## Conclusions

Geographical inequalities in accessing diagnosis and care for dementia are abundant and have been evidenced to date in 11 countries. Limited novel solutions have been identified to address rural inequalities, with early success in improving efficient diagnosis rates. However, suitable care remains limited in rural settings, but also within more urban areas, albeit generally care in urban areas appears to be more readily available, but not necessarily accessible for everyone. Early evidence also indicates higher mortality rates in people with dementia living in socio-economically deprived neighbourhoods and rural areas, albeit evidence is limited to two studies from different countries and thus lacks representativeness and requires further research. Further research needs to explore geographical inequalities for people with rarer dementia subtypes and in LMICs, as well as in relation to accessing residential care. Practical solutions to addressing geographical inequalities need to take into consideration the financial implications of having to access distant health and social care services, and may be summarised in the following practical recommendations: 1. Need for greater availability of suitable services across more rural areas; 2. Services need to meet the needs of people with different dementia subtypes, or specific subtypes, better; 3. Financial support to enable transport access to diagnosis and care settings could facilitate access; 4. Wider roll out of single-day diagnosis centres to reduce need for travel to see health care professionals when seeking a diagnosis.

## Funding

This independent research is funded by the National Institute for Health and Care Research Applied Research Collaboration North West Coast (ARC NWC). The views expressed in this publication are those of the author(s) and not necessarily those of the National Institute for Health and Care Research or the Department of Health and Social Care. This was also supported by an NIHR School for Social Care Leadership Award to the author. This research was also supported by an NIHR Undergraduate internship scheme awarded to CG, MP, and MR as supervisors. This research was also supported by NIHR/ Alzheimer Society DEMCOMM fellowships awarded to MP and MR.

## CRediT authorship contribution statement

**Polden Megan:** Writing – review & editing, Formal analysis, Conceptualization. **Carton Joan:** Writing – review & editing, Formal analysis. **Gray Annabel:** Writing – review & editing, Formal analysis, Data curation. **Godfrey Abigail:** Writing – review & editing, Formal analysis, Data curation. **Megan Rose Readman:** Writing – review & editing, Formal analysis, Conceptualization. **Clarissa Giebel:** Writing – original draft, Project administration, Methodology, Formal analysis, Conceptualization.

## Declaration of Competing Interest

None.
